# Spatial Prediction of Current and Future Flood Susceptibility: Examining the Implications of Changing Climates on Flood Susceptibility Using Machine Learning Models

**DOI:** 10.3390/e24111630

**Published:** 2022-11-10

**Authors:** Navid Mahdizadeh Gharakhanlou, Liliana Perez

**Affiliations:** Laboratory of Environmental Geosimulation (LEDGE), Department of Geography, University of Montreal, 1375 Avenue Thérèse-Lavoie-Roux, Montréal, QC H2V 0B3, Canada

**Keywords:** climate change, machine learning (ML), geographical information systems (GIS), flood susceptibility mapping, natural hazards

## Abstract

The main aim of this study was to predict current and future flood susceptibility under three climate change scenarios of RCP2.6 (i.e., optimistic), RCP4.5 (i.e., business as usual), and RCP8.5 (i.e., pessimistic) employing four machine learning models, including Gradient Boosting Machine (GBM), Random Forest (RF), Multilayer Perceptron Neural Network (MLP-NN), and Naïve Bayes (NB). The study was conducted for two watersheds in Canada, namely Lower Nicola River, BC and Loup, QC. Three statistical metrics were used to validate the models: Receiver Operating Characteristic Curve, Figure of Merit, and F1-score. Findings indicated that the RF model had the highest accuracy in providing the flood susceptibility maps (FSMs). Moreover, the provided FSMs indicated that flooding is more likely to occur in the Lower Nicola River watershed than the Loup watershed. Following the RCP4.5 scenario, the area percentages of the flood susceptibility classes in the Loup watershed in 2050 and 2080 have changed by the following percentages from the year 2020 and 2050, respectively: Very Low = −1.68%, Low = −5.82%, Moderate = +6.19%, High = +0.71%, and Very High = +0.6% and Very Low = −1.61%, Low = +2.98%, Moderate = −3.49%, High = +1.29%, and Very High = +0.83%. Likewise, in the Lower Nicola River watershed, the changes between the years 2020 and 2050 and between the years 2050 and 2080 were: Very Low = −0.38%, Low = −0.81%, Moderate = −0.95%, High = +1.72%, and Very High = +0.42% and Very Low = −1.31%, Low = −1.35%, Moderate = −1.81%, High = +2.37%, and Very High = +2.1%, respectively. The impact of climate changes on future flood-prone places revealed that the regions designated as highly and very highly susceptible to flooding, grow in the forecasts for both watersheds. The main contribution of this study lies in the novel insights it provides concerning the flood susceptibility of watersheds in British Columbia and Quebec over time and under various climate change scenarios.

## 1. Introduction

Floods have become the most prevalent natural catastrophe, accounting for 44% of all natural disasters and harming 1.6 billion people globally between 2000 and 2019 [1]. Floods are the most common natural disaster in Canada [2]. According to the Canadian Disaster Database [3], there were 241 flood disasters in Canada between 1900 and 2005, about five times as frequently as wildfires, the second most common natural hazard in Canada.

Climate change poses a significant peril to present and future generations. Climate change has made natural disasters more unpredictable, causing them to occur more frequently and with more significant impact [4]. Climate changes inducing hydrological changes and precipitation amounts affect the likelihood of flood occurrences. Accordingly, floods are more likely to occur in areas where the climate shifts toward more intense and frequent precipitation [5]. Although flood avoidance is inescapable, accurate flood forecasting, which considers the impacts of climate changes through proper models, might aid in future damage reduction.

Due to the intrinsic complexity of the flood phenomenon and the influence of various variables on floods, simple models are insufficient for accurate flood prediction [6]. In general, flood susceptibility modeling and mapping methodologies have been developed using two main types of models: physically-based and data-driven; albeit certain studies to assess flood susceptibility employed Multi-Criteria Decision Analysis (MCDA), such as the Analytical Hierarchy Process (AHP) and Analytical Network Process (ANP) [7,8,9]. The main drawback of the MCDA-based flood models is that they are prone to be distorted due to their dependence on expert knowledge [10]. 

Although physical models have shown to be capable of investigating a wide range of phenomena (e.g., rainfall-runoff [11], hydraulic models of flow [12], and flood [13]), developing physical flood-prediction models requires using fundamentally complex equations and in-depth knowledge and expertise of the flood phenomenon [14,15]. Owing to the drawbacks of the physical models, the usage of advanced data-driven models has been increasingly popular in recent decades [16,17,18]. When compared to physical models, data-driven models have three key advantages: (1) nonlinearity and numerical formulating of the flood based on historical data without requiring knowledge about the underlying physical processes, (2) providing more straightforward implementation with low computation cost, high performance, and high accuracy [19], and (3) relatively minor complexity [20].

Among the data-driven models, various ML models have been suggested and implemented to assess flood susceptibility [21,22]. The most frequently used ML models are Decision Tree (DT) [23], Random Forest (RF) [24,25], Naïve Bayes (NB) [26,27,28], Multilayer Perceptron Neural Network (MLP-NN) [29,30], Adaptive Neuro-Fuzzy Inference System (ANFIS) [31,32], Support Vector Machine (SVM) [33], Gradient Boosting Machine (GBM) [34], Fuzzy Logic [35], etc. Although there is no consensus on which method or group of methodologies may produce the most accurate predictions [36], ML models have recently successfully assessed flood susceptibility with greater accuracy [37,38].

Flood susceptibility is described as a quantitative or qualitative assessment of a place with geographical distribution of floods and a high likelihood of flooding [39]. Flood susceptibility maps illustrate the susceptibility of places to flooding and highlight locations that are prone to flooding. Enhancing the accuracy of flood susceptibility maps is a concern for flood disaster management researchers and decision-makers. Flood susceptibility maps become more practical to local governments and policy-makers as flood estimations get more precise. In recent years, advances in data collection and preparation methods using RS and GIS have led to an increase in the reliability and accuracy of flood prediction models, and consequently flood susceptibility mapping [40,41,42]. RS provides a variety of data sources, data with excellent quality, day and night data gathering capabilities, and rapid analysis [43,44], and GIS is designed for the storage, retrieval, and analysis of geographically referenced data [45].

Numerous studies have been conducted using ML models to evaluate the impacts of climate change on the flood susceptibility [46,47,48,49,50]. However, ML models of MLP-NN, RF, NB, and GBM have not yet been used to investigate the impacts of climate change on the flood susceptibility for two watersheds in Canada, namely, Lower Nicola River, BC and Loup, QC. In doing so, we investigated the impacts of climate changes for the years of 2020, 2050, and 2080 under three different climate change scenarios of RCP2.6 (i.e., optimistic scenario), RCP4.5 (i.e., business as usual scenario), and RCP8.6 (i.e., pessimistic scenario) to contribute towards a dynamic estimation of flood susceptibility in these areas. Moreover, another highlight of the present study was to consider various topographic, hydrologic, environmental, and geologic flood conditioning factors yield by means of RS and GIS techniques.

## 2. Materials and Methods

### 2.1. Description of the Study Areas

**Figure 1:**
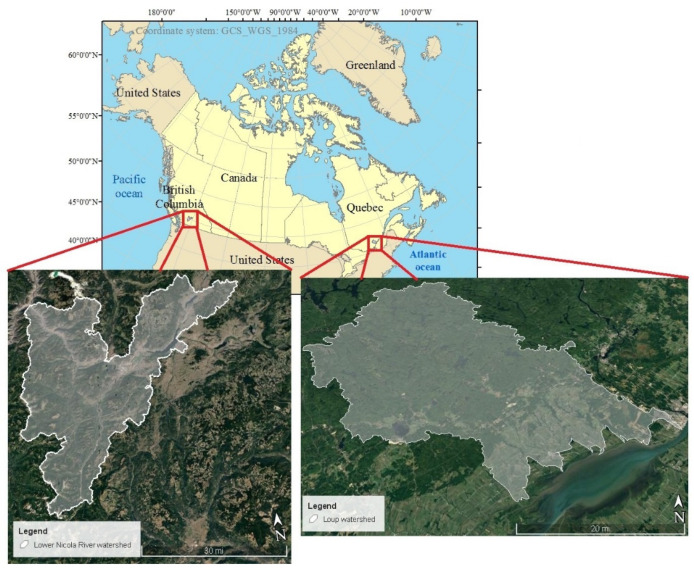
The geographical location of study watersheds, namely Loup, QC and Lower Nicola River, BC.

This study focuses on two watersheds, one in Quebec (QC) and the other in British Columbia (BC) provinces. On 3 May 2017, Eastern Canada was flooded due to heavy rain, with QC being the worst hit. The Loup watershed located in QC was one of the watersheds affected by the May 2017 flood. Likewise, the watershed of the Lower Nicola River, located in south-central BC province, was struck by a flood triggered by heavy rain on 14 November 2021. The location of both watersheds was depicted in Figure 1.

**Figure 2:**
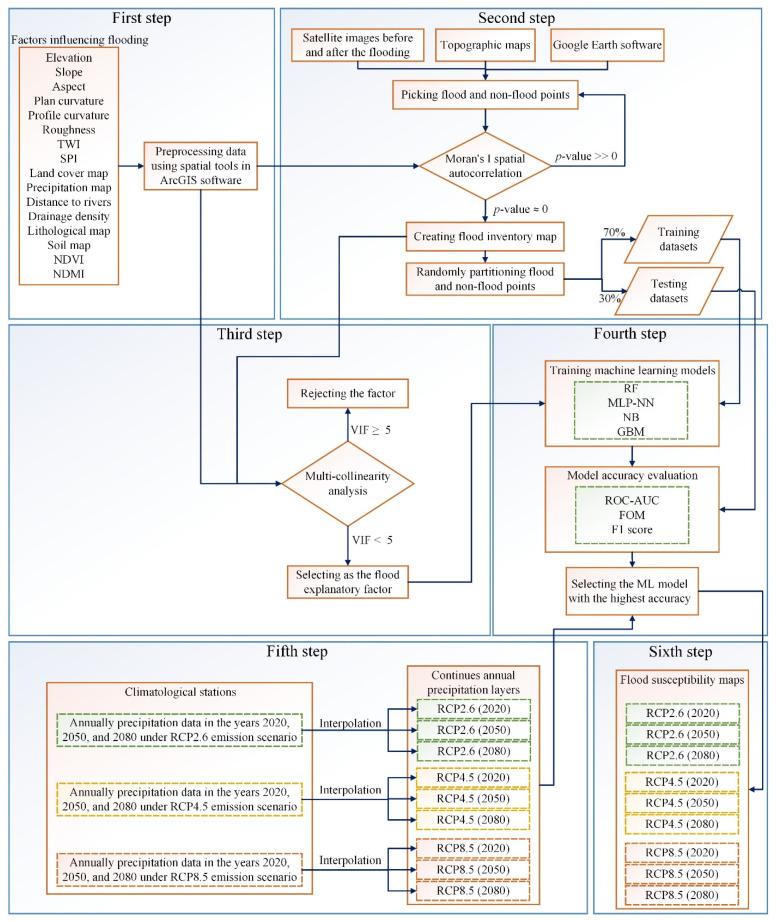
The methodology flowchart of research.

This study used four flood susceptibility assessment models to predict flood susceptibility in two different study regions correctly. The primary modeling technique for this investigation was broken down into six significant steps: (i) gathering and preparing the factors influencing flooding, (ii) iteratively picking flood and non-flood points in study areas and calculating Moran’s I spatial autocorrelation for all factors; the set of points which the P-value for all factors was obtained extremely close to zero and less than the threshold (i.e., 0.05) was chosen to create the flood inventory map, (iii) assessing the correlation between flood occurrence and flood influencing factors using multicollinearity analysis and either including or excluding them in the following processes, (iv) training the ML models, evaluating and comparing their performance using three statistical metrics, and choosing the model with the highest accuracy, (v) gathering and preparing the annual precipitation data in three years of 2020, 2050, and 2080 under three climate change scenarios (i.e., RCP2.6, RCP4.5, and RCP8.5), and (vi) providing associated flood susceptibility maps with the years and scenarios. The methodology flowchart of the research was shown in Figure 2.

### 2.2. Flood Inventory Maps

**Figure 3:**
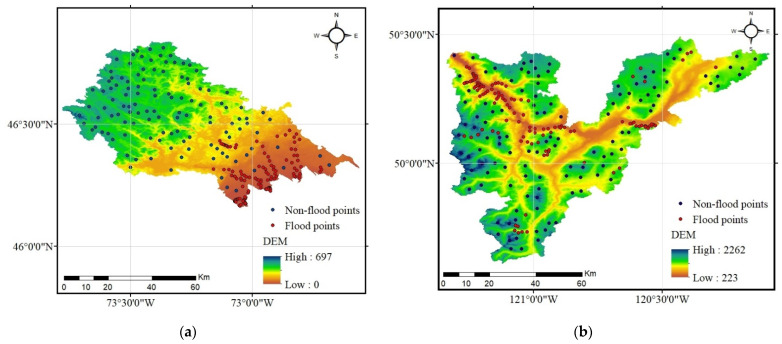
Flood inventory maps: (**a**) Loup watershed, and (**b**) Lower Nicola River watershed.

The flood inventory map depicts the flooded and non-flooded locations that are used to train ML models. The basic premise behind the flood inventory map is that future floods will follow the same pattern as previous floods. The flood inventory map can be produced by field survey, satellite images before and after the flooding, topographic maps, and Google Earth software. Accordingly, having performed the Moran’s I spatial autocorrelation analysis for all flood influencing factors in the points picked, the flood inventory map was provided for each study watershed by ascertaining 120 flood sites using data from previous floods collated from satellite images, topographic maps, and Google Earth software in both watersheds (Figure 3). It is worth mentioning that the flood inventory maps created for the Loup and Lower Nicola River watersheds were a compilation of a single flood event that occurred on 3 May 2017, and 14 November 2021 respectively. Although, no specific literature on the number of flood-present and flood-absent places has been discovered, an approximately equal number of them is preferred for flood susceptibility mapping [27,51]. Accordingly, 120 non-flood sites were picked randomly in both watersheds, to establish a dichotomous dependent variable for modeling. Following the earlier studies [5,52], random selection was used to split two datasets into training (70%) and testing (30%) sets.

### 2.3. Flood Explanatory Factors and Their Preparation Processes

The intensity and severity of floods majorly rely on topographic, hydrologic, environmental, and geologic factors [53]. Accordingly, the sixteen factors reflecting topographic, hydrologic, environmental, and geologic attributes were recognized, and the data that describe each flood explanatory factor was compiled for both watersheds. Concerning a thorough review of the related literature [5,54,55,56], the following sets of factors were chosen: elevation, slope, aspect, plan curvature, profile curvature, roughness, Topographic Wetness Index (TWI), land cover, precipitation, distance from rivers, drainage density, lithology, soil, Stream Power Index (SPI), Normalized Difference Vegetation Index (NDVI), and Normalized Difference Moisture Index (NDMI). 

The influences of topographic-related factors (i.e., elevation, slope, aspect, curvature, SPI, TWI, and roughness) derived from the digital elevation model (DEM) on flood occurrences have been well recognized in the literature [5,57,58]. The effects of elevation on hydrology and floods are significant [59]. Floods are uncommon in high-elevation places, while runoff gathers from above at lower altitudes, making floods more prevalent [60]. The DEM with a spatial resolution of 30 m was produced through Shuttle Radar Topography Mission (SRTM) and clipped to the study areas’ border. Slope determines the rates of surface runoff [61]. Plan and profile curvatures reveal the concavity and convexity of slopes influence on flow velocity [5]. The aspect factor is connected with water flow convergence and directions [56]. The slope, aspect, plan and profile curvature layers were provided by applying the spatial analyst tools of Slope, Aspect, and Curvature on the DEM, respectively. SPI and TWI are also two common topographical factors affecting flow intensity and water accumulation [62]. The SPI and TWI were also obtained from the DEM layer using the Raster Calculator tool according to Equations (1) and (2), respectively.

(1) SPI=α∗tanβ

(2) $$TWI=\ln\left(\frac{\alpha }{\tan\beta }\right)$$

Here, α denotes the cumulative upstream discharge at one point, or flow accumulation ($m^{2}m^{-1}$), and β is the slope (in radian). 

Roughness which indicates the elevation differences between neighboring pixels, is another factor affecting the surface runoff [63]. To generate the roughness layer from the DEM layer, the Focal Statistics tool was used three times to acquire the mean, minimum, and maximum focal statistics layers. Then, the Raster Calculator tool was applied to them using Equation (3).

(3) $$Roughness=(FS_{mean}-FS_{min})/(FS_{max}-FS_{min})$$

Here, $FS_{mean}$, $FS_{min}$, and $FS_{max}$ represent the mean, minimum, and maximum focal statistical layer, respectively.

Another factor influencing the likelihood of flooding is stream density, which measures how much of a watershed is drained by stream channels [64]. Having prepared the stream layers from DEM using the Hydrology tools, the Line Density tool was used to obtain the stream density layer. Concerning the earlier studies [65,66], the likelihood of flooding is also influenced by proximity to a river. As the distance from rivers decreases, the chance of flooding increases. To acquire the layer of distance from rivers, the Euclidean Distance tool was applied to the river polyline shapefile. Soil and land cover are also influencing factors due to their influence on the infiltration and runoff speed [67,68]. Likewise, geology which indicates underlying rock types impacts infiltration and runoff in watersheds [38]. To obtain the Soil, Land cover, and geology maps for the study areas, having downloaded the layers, they were clipped to the study areas’ border. Precipitation is also a hydrologic factor that significantly influences the incidence of floods [69]. To provide continuous layers of average annual precipitation, first, annual precipitation was gathered at 10 climatological stations (both inside and outside the watersheds) for the period 2000–2020. After calculating the average annual precipitation at climatological stations, the Ordinary Kriging interpolation method was used. Vegetation, which on one hand impacts the evaporation process and hydrological cycle while also acting as a barrier to the flow of water over the ground, has a substantial impact on run-offs and floods. Accordingly, NDMI, a metric indicating the moisture content of vegetation, was employed in the modeling process [70]. There is also an inverse relationship between vegetation density and floods [34]. NDVI, an essential metric representing vegetation coverage, was also considered in the modeling process [71]. The NDMI and NDVI layers in the study area were obtained from the Landsat 8 Operational Land Imager (OLI) and Thermal Infrared Sensor (TIRS) satellite images using the Raster Calculator according to Equations (4) and (5), respectively.

(4) $$NDMI=\frac{NIR-SWIR}{NIR+SWIR}$$

(5) $$NDVI=\frac{NIR-R}{NIR+R}$$

Here, *NIR* is the Near Infrared band (band 5 of Landsat 8), *SWIR* is the Short-Wave Infrared band (band 6 of Landsat 8), and *R* is the Red band (band 4 of Landsat 8).

**Figure A1:**
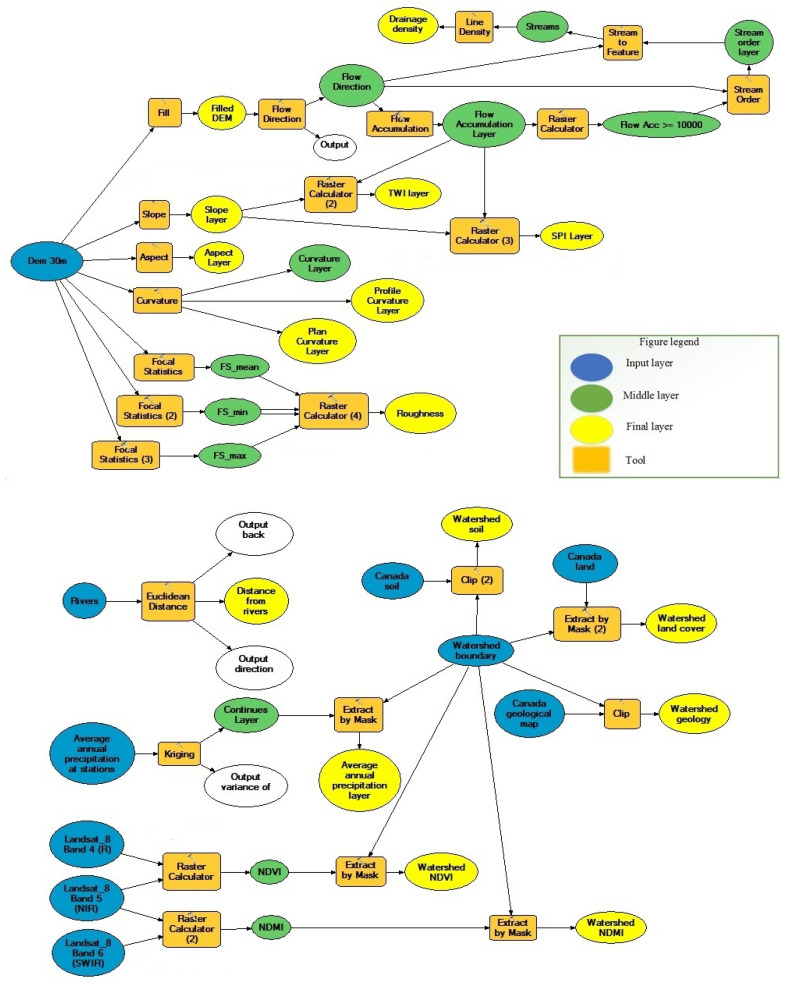
The overall data preparation flowchart using the Model Builder extension of ArcGIS.

**Figure A2:**
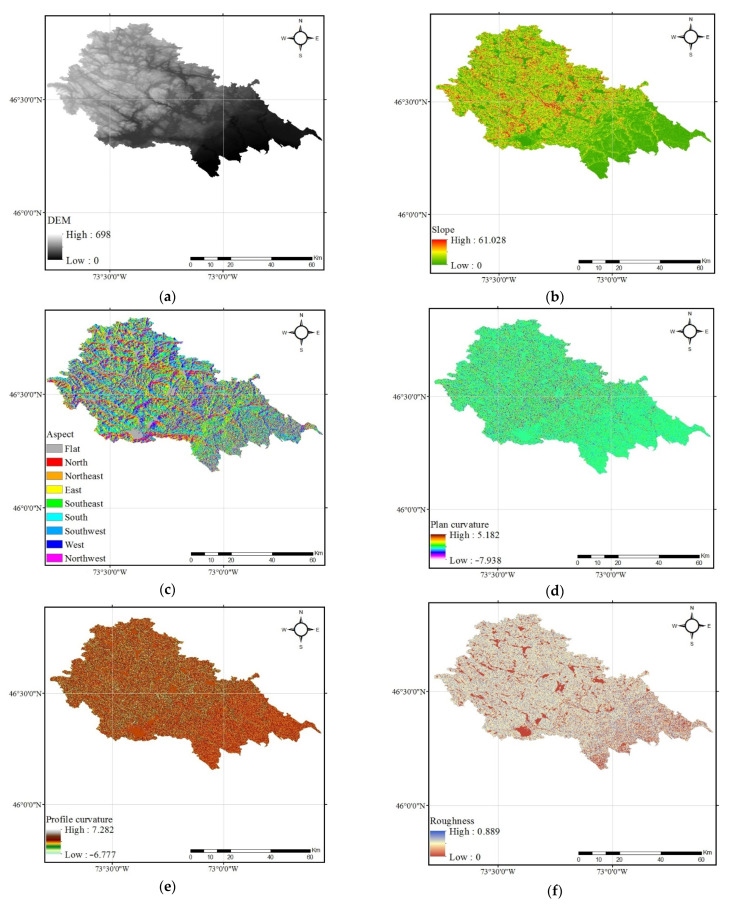

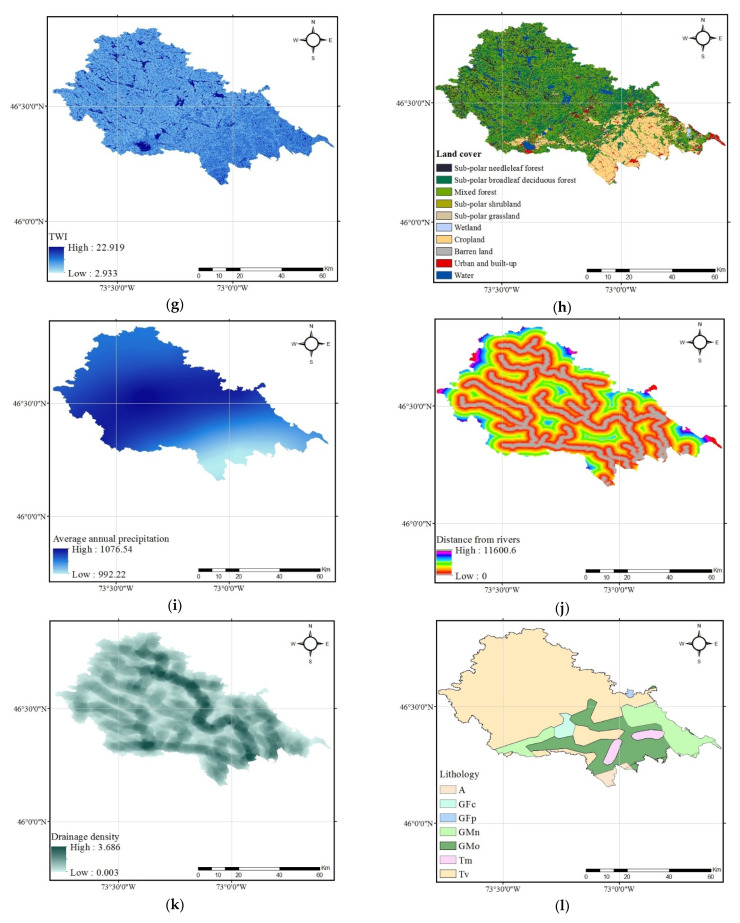

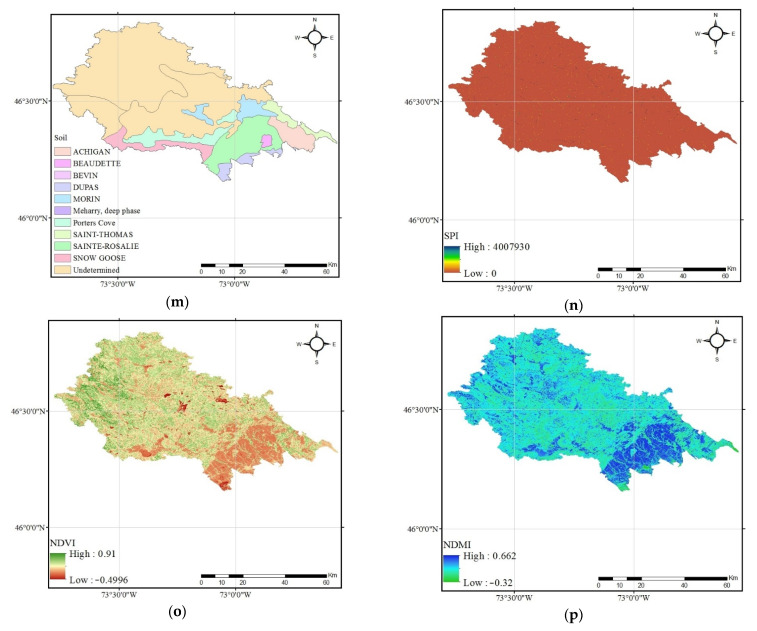
Thematic maps of flood explanatory factors in the Loup watershed: (**a**) DEM, (**b**) slope, (**c**) aspect, (**d**) plan curvature, (**e**) profile curvature, (**f**) roughness, (**g**) TWI, (**h**) land cover, (**i**) precipitation, (**j**) distance from rivers, (**k**) drainage density, (**l**) lithology, (**m**) soil, (**n**) SPI, (**o**) NDVI, (**p**) NDMI.

**Figure A3:**
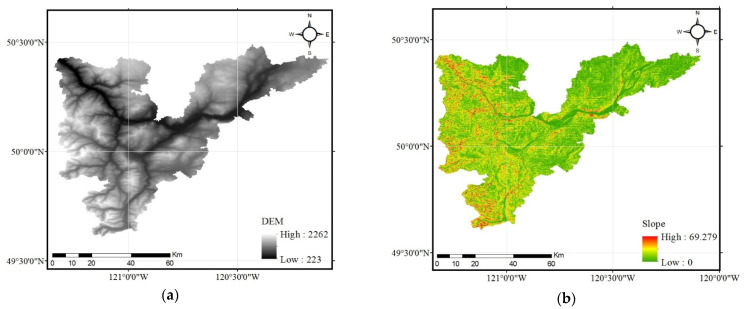

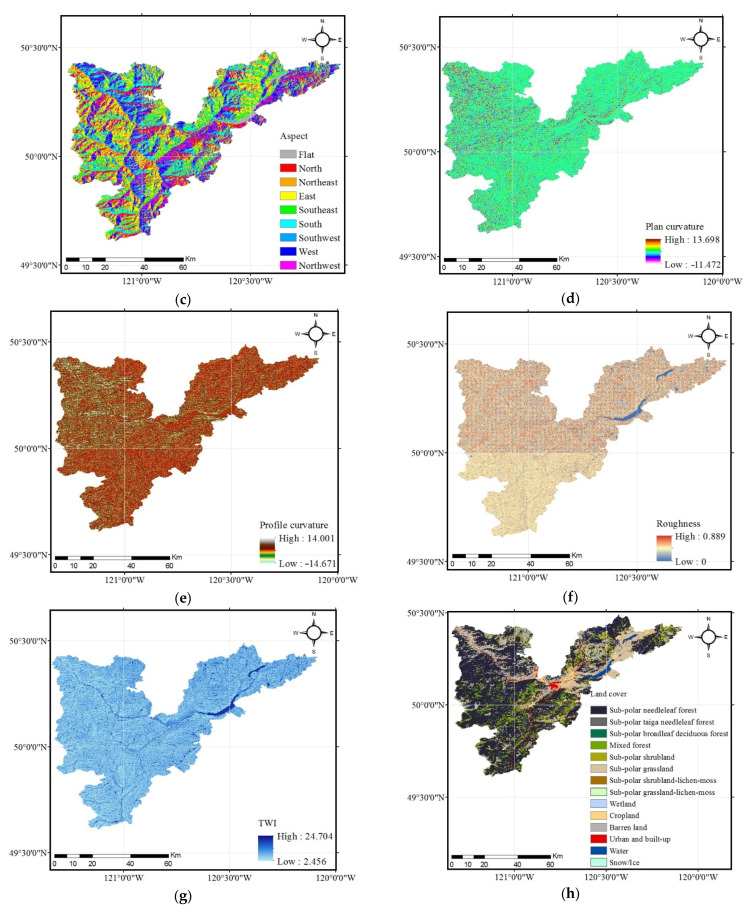

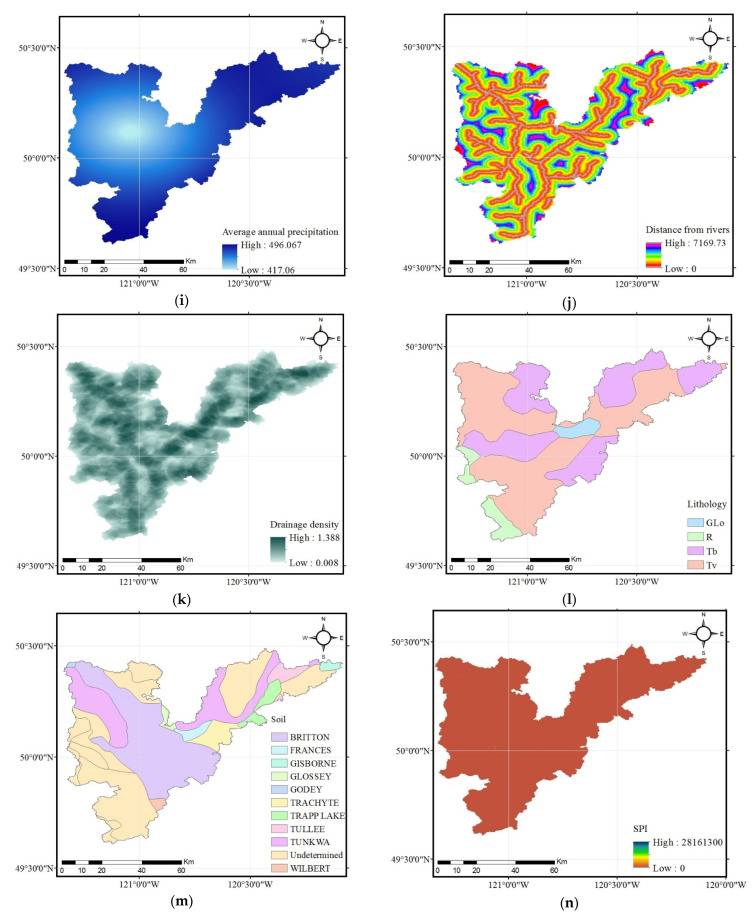

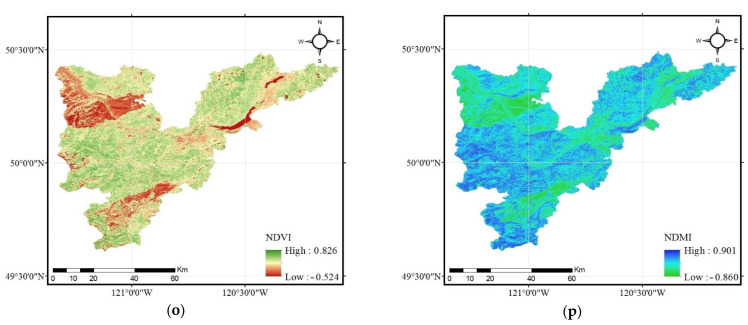
Thematic maps of flood explanatory factors in the Lower Nicola River watershed: (**a**) DEM, (**b**) slope, (**c**) aspect, (**d**) plan curvature, (**e**) profile curvature, (**f**) roughness, (**g**) TWI, (**h**) land cover, (**i**) precipitation, (**j**) distance from rivers, (**k**) drainage density, (**l**) lithology, (**m**) soil, (**n**) SPI, (**o**) NDVI, (**p**) NDMI.

All flood explanatory data acquired for this investigation, along with their sources, were summarized in Table 1. The overall data preparation flowchart was given in Figure A1 (Appendix A). Moreover, each flood explanatory factor was plotted on a map after the preparation processes (Figure A2 and Figure A3, Appendix B). All the factors were designed to have a comparable spatial scope of 30 m pixel size due to the spatial resolution of the land cover data (i.e., 30 m).

### 2.4. Multicollinearity of Flood Explanatory Factors

Before implementing the models, a multicollinearity investigation of the independent variables is indispensable to reduce the risk of inaccuracy in flood susceptibility models. The multicollinearity analysis investigates whether the variables are affected by multicollinearity. In doing so, multicollinearity involves tightly coupling many independent variables in a multiple regression model and removing variables with significant collinearity. Variance inflation factors (VIF) and tolerance (TOL) are two exponents frequently used to analyze the multicollinearity of variables.

Table 2 shows the VIF and TOL calculated values for the proposed flood influencing factors in multicollinearity analysis in both watersheds. The TOL values less than 0.1 or VIF values greater than 10 indicate the multi-collinearity issue [72]. However, the threshold of 5 for VIF was taken into consideration in this study to choose significant independent predictors with a high degree of certainty. Accordingly, except for DEM factor in the Loup watershed, the rest of the 15 explanatory variables were allowed for usage throughout the modeling process. In the Lower Nicola River watershed, on the other hand, the multicollinearity statistics indicated that all 16 explanatory variables could be included in the modeling process.

### 2.5. Predicting Future Precipitation Data

Many variables impact climate, most notably human activity, and greenhouse gas emissions. Although there are considerable uncertainties in climate forecasts due to the complex nature of the climate system, greenhouse gas emissions, and human activities, some aspects of this variability are thought to be predictable for a decade or more in advance. Emissions scenarios are one method of presenting a variety of possible futures depending on various future emissions. Accordingly, a collection of scenarios known as Representative Concentration Pathways (RCPs) is frequently used to investigate future climate change. RCPs are intended to offer probable future human emission trends. These include considering future greenhouse gas emissions, deforestation, population growth, and a variety of other factors. Based on best practices in the global science community, the Government of Canada typically offers three RCPs: RCP8.5 (high global emission scenario), RCP4.5 (medium global emission scenario), and RCP2.6 (low emission global scenario) [73].

In this study, Coupled Model Intercomparison Project Phase 5 (CMIP5) climate model datasets which were downscaled and bias-adjusted using the BCCAQv2 method were utilized. The preparation of future precipitation data was carried out in two steps: first, the annual precipitation data under three emission scenarios of RCP2.6 (optimistic scenario), RCP4.5 (business as usual scenario), and RCP8.5 (pessimistic scenario) were collected (from https://climatedata.ca/ (accessed on 1 March 2022)) at climatological stations inside and outside of the study areas in the years 2020, 2050, and 2080; then, the annual precipitation amounts at stations were interpolated in ArcGIS using the Ordinary Kriging interpolation method to provide continuous annual precipitation layers for all three scenarios. Having prepared the precipitation layers that corresponded with each scenario for the years 2020, 2050, and 2080, they were used in the ML models to provide the associated flood susceptibility maps.

### 2.6. Methods for Flood Susceptibility Modeling

#### 2.6.1. Multilayer Perceptron Neural Network (MLP-NN)

MLP-NN is classified as a feed-forward neural network trained using supervised and back-propagation learning methods. MLP-NN has been widely employed as a benchmark model in a variety of studies owing to its capabilities in the prediction and modeling of nonlinear and complicated phenomena [74,75]. Basically, the MLP-NN model comprises a system of simply interconnected neurons that are organized into three layers: an input layer, one or more hidden layers, and finally, an output layer. Neurons in each layer receive values, which are multiplied by corresponding weights, then summed up and passed through a nonlinear function (i.e., activation function) [76]. Using the activation function on the weighted sum enables the MLP-NN to account for the nonlinear relationship between the independent and dependent variables [77]. Accordingly, the MLP-NN model estimates the nonlinear connections between the independent variables (i.e., flood explanatory factors) and the dependent variable (i.e., flood occurrences).

**Figure 4:**
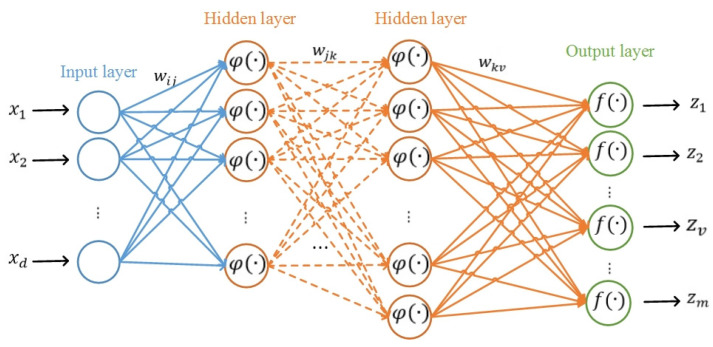
The structure of the MLP-NN model.

The neurons in two sequential layers are linked by the unknown weights whose values are estimated through the iterative back-propagation learning technique. The back-propagation approach is generally an iterative gradient-based learning technique (e.g., Stochastic Gradient Descent (SGD)) that aims to reduce the discrepancy between the outputs of the network and actual target values (i.e., reduce the value of the cost function) by estimating the weights in each iteration [76]. The architecture of an MLP-NN was shown in Figure 4. The connections of the nodes from different layers are made using Equations (6)–(8).

(6) $$t_{j}=\varphi \left(\overset{d}{\underset{i=1}{\sum }}w_{ij}x_{i}+w_{0j}\right)$$

(7) $$y_{k}=\varphi \left(\overset{n_{H_{1}}}{\underset{j=1}{\sum }}w_{jk}t_{j}+w_{0k}\right)$$

(8) $$z_{v}=f\left(\overset{n_{H_{2}}}{\underset{k=1}{\sum }}w_{kv}y_{k}+w_{0v}\right)$$

Here, presuming the MLP-NN made up of two hidden layers, $d,\;n_{H_{1}},$ and $n_{H_{2}}$ are the number of nodes in the input, first hidden, and second hidden layer, respectively. $w_{ij},\;w_{jk}$, and $w_{kv}$ are the connection weights between two nodes from two consecutive layers. Moreover, $w_{0j},$ $w_{0k},$ and $w_{0v}$ are the intercepts of the input, first hidden and second hidden layer, respectively. $x_{i}$, $t_{j},$ $y_{k},$ and $z_{v}$ are the nodes in each input layer, first hidden, second hidden, and output, respectively. φ is the activation function of all layers except the output layer, and f is the activation function of the output layer.

#### 2.6.2. Naïve Bayes (NB) Model

NB methods are a set of supervised learning algorithms based on Bayes’ Theorem and the assumption of conditional independence between every pair of features. In other words, an NB classifier posits that the existence of one feature in a class is independent of the presence of any other feature. The Bayes theorem proposes a method for computing posterior probability $P(c|x_{1},x_{2},\cdots ,x_{d})$ from P(c), $P\left(x_{1},x_{2},\cdots ,x_{d}\right)$, and $P(x_{1},x_{2},\cdots ,x_{d}|c)$ (Equation (9)) given the naive conditional independence assumption (Equation (10)) [78].

(9) $$P\left(c|x_{1},x_{2},\cdots ,x_{d}\right)=\frac{P\left(x_{1},x_{2},\cdots ,x_{d}|c\right)*P\left(c\right)}{P\left(x_{1},x_{2},\cdots ,x_{d}\right)}$$

(10) $$P\left(x_{1},x_{2},\cdots ,x_{d}|c\right)=\overset{d}{\underset{i=1}{\prod }}P\left(x_{i}|c\right)=P\left(x_{1}|c\right)*P\left(x_{2}|c\right)*\cdots *P(x_{d}|c)$$

Here, $P(c|x_{1},x_{2},\cdots ,x_{d})$ is the posterior probability of class (*c*, target) given features, ($x_{1},x_{2},\cdots ,x_{d}$), P(c) is the prior probability of class, $P(x_{1},x_{2},\cdots ,x_{d}|c)$ is the likelihood which is the probability of predictor given class, and $P\left(x_{1},x_{2},\cdots ,x_{d}\right)$ is the prior probability of predictor.

The Gaussian NB method was chosen from among the various types of NB methods (e.g., Gaussian, Multinomial, Complement, Bernoulli) owing to its common use in classification. The Gaussian NB model assumes that features follow a normal distribution, and the likelihood of the features is calculated according to Equation (11). The parameters $\sigma_{c}$ and $\mu_{c}$ in Equation (11) are estimated using maximum likelihood.

(11) $$P\left(x_{i}|c\right)=\frac{1}{\sqrt{2\pi \sigma_{c}^{2}}}\exp\left(-\frac{{\left(x_{i}-\mu_{c}\right)}^{2}}{2\sigma_{c}^{2}}\right)$$

#### 2.6.3. Random Forest (RF)

RF is one of the popular ML algorithms for addressing multi-classification and prediction issues [79]. The RF technique is a collection of DTs used to predict categorization or regression. The main procedure of the RF algorithm is to (1) resample the original data set using bootstrap (i.e., sampling with replacement) to generate various subsets with sizes equal to the original set, (2) use the subsets to construct DTs, and (3) combine the prediction or classification results of all the decision trees to obtain the final results [80]. One of the significant issues with DTs is that they are highly sensitive to training data and tend to over-fit the training detests, consequently, perform poorly when an unknown dataset is given. Using the RF method to address this flaw is a viable option. Accordingly, a portion of the input records, as well as features, were picked at random, and DTs were created according to each set of inputs and features chosen.

#### 2.6.4. Gradient Boosting Machine (GBM)

GBM is a supervised machine learning approach for classification and regression problems that use a prediction model in the form of an ensemble of weak prediction models. The central notion underlying it is a model built from a set of weak learners, commonly decision trees (DTs). The GBM is similar to functional gradient descent in that it applies a new learner to residual errors created by the prior learner to minimize a loss at each gradient descent step [81]. As with other boosting methods, various loss functions might be considered. The constructed decision tree is optimized with the gradient boosting approach in this model. Gradient boosting approaches create the solution and address the over-fitting problem by maximizing the loss functions in a stage-wise structure [82]. Presuming a custom base-learner *h*(*x*,*θ*) (such as a decision tree) and a loss function Ψ(y,f(x)); directly estimating the parameters is challenging; hence, an iterative model is recommended. The model will be updated, and *h*(*x*,*θ*) will be chosen as the new base-learner feature, with the *t* increment driven by Equation (12) [81].

(12) $$g_{t}\left(x\right)=E_{y}{[\frac{\partial \Psi \left(y,f\left(x\right)\right)}{\partial f\left(x\right)}|x]}_{f\left(x\right)={\hat{f}}^{t-1}\left(x\right)}$$

Instead of searching the function space for a general solution for the boost increment, one may just select the new function increment that is the most correlated with $g_{t}\left(x\right)$. This replaces the hard optimization problem with the standard least-squares optimization problem according to Equation (13) [81].

(13) $$\left(p_{t},\theta_{t}\right)=arg\;min_{p,\theta }\overset{N}{\underset{i=1}{\sum }}{[-g_{t}\left(x_{i}\right)+\rho h\left(x_{i},\theta \right)]}^{2}$$

The procedure of GBM algorithms include: (i) presuming that $\hat{f_{0}}$ is constant, (ii) calculating $g_{t}\left(x\right)$ and training *h*($x_{i}$,θ) function, and (iii) finding element $\rho_{i}$ and updating the function $\hat{f_{i}}={\hat{f}}_{i-1}+\rho_{i}h\left(x_{i},\theta \right)$.

### 2.7. Model Evaluation Metrics

#### 2.7.1. Receiver Operating Characteristic (ROC) Curve

A ROC curve is a graphical plot that illustrates the diagnostic ability of a binary classifier system as its discrimination threshold is varied. The ROC curve is a popular method for assessing the performance of predictive models [83]. This accuracy criterion has been increasingly utilized in a variety of susceptibility mapping applications using ML models, including landslide [84,85], earthquake [86,87], and flood [88,89,90]. For quantitative evaluation, this method employs the area under the ROC curve (AUC), which plots false positive rate (*FPR*) (Equation (14)) on the x-axis against true positive rate (*TPR*) (Equation (15)) on the y-axis [91]. A higher AUC value depicts a better goodness-of-fit of the model. Generally, AUC values ranging from 0.8 to 0.9 indicate extremely strong performance for the prediction model [88].

(14) $$FPR=1-Specificity=\frac{FP}{FP+TN}$$

(15) $$TPR=Sensitivity=\frac{TP}{TP+FN}$$

Here, *TP* (true positive) and *TN* (true negative) are test results that correctly indicate the presence and absence of a condition or characteristics, respectively. *FP* (false positive) and *FN* (false negative) are test results that incorrectly indicate the presence and absence of a condition or characteristics, respectively.

#### 2.7.2. Figure of Merit (FOM)

The *FOM* is a statistical measure of sample set similarity and diversity. Equation (16) is used to calculate the *FOM*, which is the equivalent of the Jaccard index. The *FOM* has a value range of 0 to 1, with 1 being the ideal match [92].

(16) $$FOM=\frac{TP}{TP+FP+FN}$$

#### 2.7.3. F1 Score

In binary classification statistical analysis, the F-score measures a test’s accuracy. The accuracy and recall (or sensitivity) of the test are used to calculate it, with precision equaling the number of true positive results divided by the total number of positive results, including those that were incorrectly identified. The harmonic mean of accuracy and recall is the F1 score (also known as the Dice similarity coefficient). The maximum possible F-score is 1, which indicates flawless accuracy and recall, while the lowest possible F-score is 0 if neither precision nor recall is zero. F1 score is calculated according to Equation (17) and auxiliary Equations (18) and (19) [93].

(17) $$F_{1}=\frac{2*precision*recall}{precision+recal}$$

(18) $$precision=\frac{TP}{TP+FP}$$

(19) $$recall=\frac{TP}{TP+FN}$$

## 3. Results

### 3.1. Model Validation and Performance Assessment

Before evaluating the findings, it is indispensable to determine the optimal values of various hyper-parameters in each ML model. Accordingly, to designate the ideal values for hyper-parameters and reduce the over-fitting issues, the 5-fold cross-validation method was used. Having employed the 5-fold cross-validation method, the ROC-AUC values for the training and validation dataset (Table 3) were calculated, considering the ideal values of corresponding hyper-parameters in each ML model.

**Figure 5:**
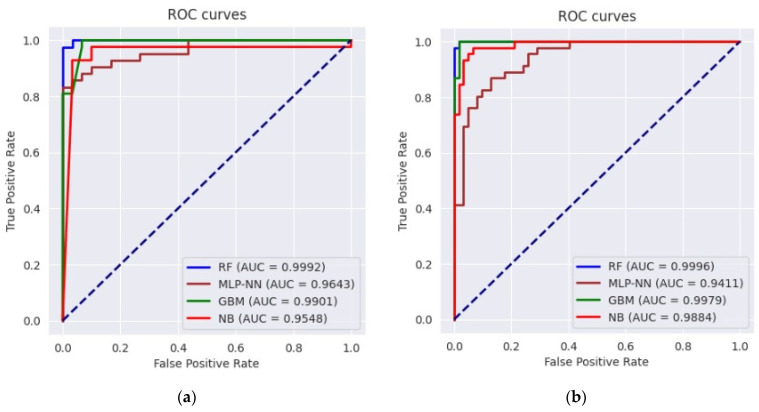
ROC curves along with the AUC values for each ML model: (**a**) the Loup watershed and (**b**) the Lower Nicola River watershed.

Three accuracy metrics of ROC-AUC, FOM, and F1 score were utilized to quantitatively measure and compare the efficiency of ML models in assessing flood susceptibility and validate the models (Table 4). It is worth emphasizing that these performance indicators were used to evaluate the models’ spatial distribution performance since they reflect the degree to which the observed flood points overlap the flood susceptibility. Even though the differences in performance between the RF and GBM models were insignificant, the RF model outperformed the GBM model with a very trifle difference given three accuracy metrics. Moreover, the models were ranked in order of performance from the best to the worst: RF, GBM, NB, and MLP-NN. The ROC curves were also plotted in Figure 5 to evaluate and compare the classifiers’ quality independent of the threshold.

### 3.2. Flood Susceptibility Map

**Figure 6:**
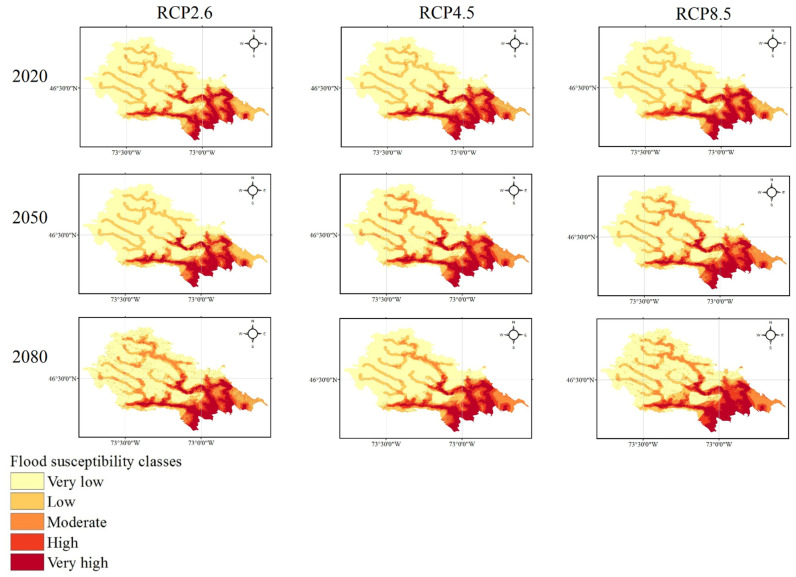
The flood susceptibility maps of the Loup, QC watershed in the years 2020, 2050, and 2080 under three climate change scenarios.

**Figure 7:**
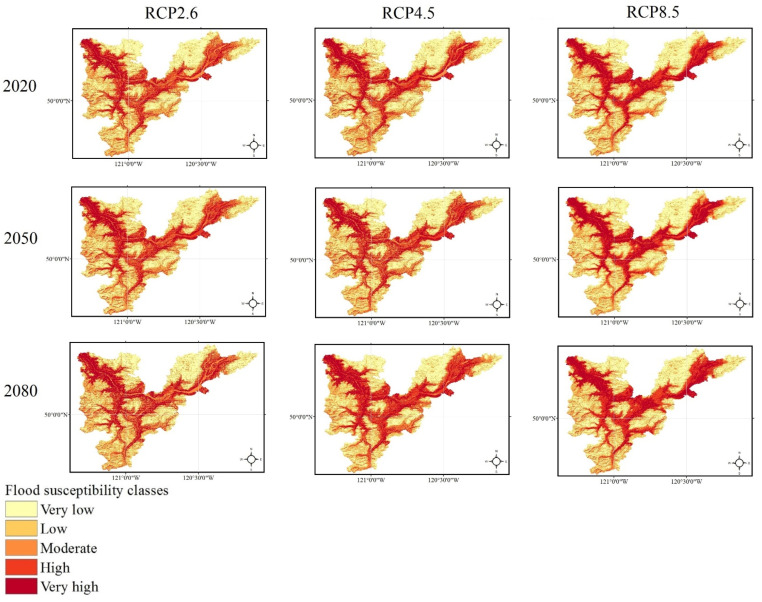
The flood susceptibility maps of the Lower Nicola River, BC watershed in the years 2020, 2050, and 2080 under three climate change scenarios.

**Figure 8:**
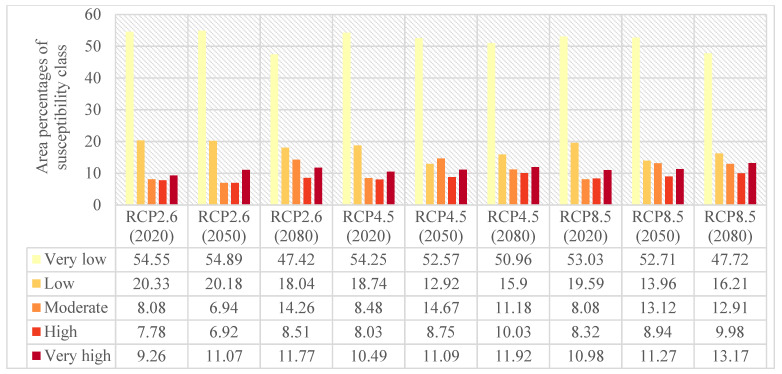
Area percentages of flood susceptibility classes under three climate change scenarios of RCP2.6, RCP4.5, and RCP8.5 in the Loup watershed.

**Figure 9:**
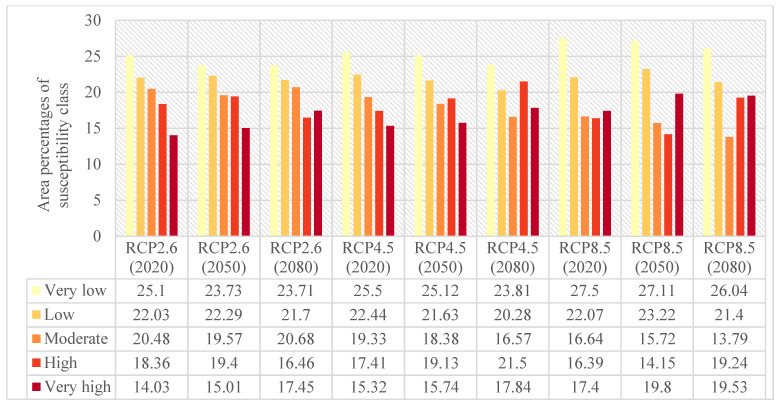
Area percentages of flood susceptibility classes under three climate change scenarios of RCP2.6, RCP4.5, and RCP8.5 in the Lower Nicola River watershed.

Providing the flood susceptibility maps and investigating the impacts of climate change on flood susceptibility of study areas throughout time were the primary objectives of this study. Accordingly, the flood susceptibility maps were mapped for both Loup (Figure 6) and Lower Nicola River (Figure 7) watersheds in the years 2020, 2050, and 2080 under three climate change scenarios of RCP2.6, RCP4.5, and RCP8.5. As briefly explained in Section 2.5, to provide flood susceptibility maps under climate change scenarios, the former precipitation layer was replaced with the intended climate change scenario precipitation layer; then, the intended precipitation layer was fed the ML model along with the rest flood explanatory factors. Finally, the resulted flood susceptibility values were stratified into five classes from very low to very high susceptibility using the natural break classification technique in ArcGIS 10.8 software. The area percentages of each flood susceptibility class given the year and climate change scenario were calculated for both the Loup watershed (Figure 8) and the Lower Nicola River watershed (Figure 9).

Considering the scenario RCP4.5 as the baseline scenario, the derived flood susceptibility map of the Loup watershed in the year 2020 revealed that the flood susceptibility was very low in 54.25% of the Loup watershed, low in 18.74%, moderate in 8.48%, high in 8.03%, and very high in 10.49%. Likewise, the resulted flood susceptibility map for the Lower Nicola River watershed in the year 2020 under the scenario RCP4.5 indicated that approximately 25.5%, 22.44%, 19.33%, 17.41%, and 15.32% of the watershed were in very low, low, moderate, high, and very high susceptibility classes, respectively. Assuming no changes in the emission scenario in the following years, in the year 2050, the flood susceptibility will be very low, low, moderate, high, and very high in 52.57%, 12.92%, 14.67%, 8.75%, 11.09% of the Loup watershed, respectively, and in the year 2080, in 50.96%, 15.9%, 11.18%, 10.03%, 11.92% of the watershed, respectively. Likewise, the area percentages of flood susceptibility classes for the Lower Nicola River in the year 2050 will be 25.12%, 21.63%, 18.38%, 19.13%, 15.74%, respectively, and in the year 2080 will be 23.81%, 20.28%, 16.57%, 21.5%, and 17.84%, respectively. Following the changes in the flood susceptibility classes in the Loup watershed, it can be concluded that irrespective of some fluctuations, the overall trend of changes is decreasing for the area percentages of very low and low flood susceptibility classes and increasing for the area percentages of the moderate, high, and very high flood susceptibility classes. As a result, the area percentages of the very low and low flood susceptibility classes were lowered and added to the area percentages of the moderate, high, and very high flood susceptibility classes, indicating that the flood susceptibility of the Loup watershed worsens over time. Similarly, the trend of changes in the flood susceptibility classes in the Lower Nicola River watershed was decreasing in the very low, low, and moderate classes and increasing in the high and very high classes, indicating that the flood susceptibility of the Lower Nicola River watershed worsens over time, as well.

Comparing the area percentages of flood susceptibility classes in both watersheds revealed that the area percentages of the Loup watershed in the moderate, high, and very high flood susceptibility classes were relatively small compared to the area percentages of the same flood susceptibility classes in the Lower Nicola River watershed. This indicates flooding is more likely in the Lower Nicola River watershed than in the Loup watershed. Moreover, it can be concluded that the most flood-prone areas in the Loup watershed were in the southern and southeast, whereas in the Lower Nicola River watershed, the most flood-prone regions were in the center, northeast, and northwest. Furthermore, concerning the area percentages of flood susceptibility classes in both watersheds and their corresponding precipitation amounts, it can be concluded that despite the relatively high precipitation amounts in the Loup watershed compared to the Lower Nicola River watershed, significantly larger area of the Lower Nicola River watershed was susceptible to flooding. As a result, our findings indicated that climatological flood explanatory factors single-handedly are inadequate in identifying flood-prone regions and that topographic, hydrologic, environmental, and geologic factors must be considered and investigated in addition to them.

**Figure 10:**
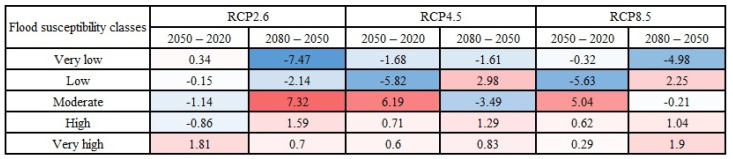
Changes (in %) in the area percentages of flood susceptibility classes based on the years in the Loup, QC watershed.

**Figure 11:**
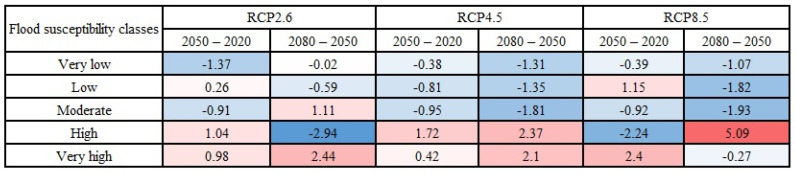
Changes (in %) in the area percentages of flood susceptibility classes based on the years in the Lower Nicola River, BC watershed.

The magnitude and direction of changes in the area percentages of flood susceptibility classes over three years (i.e., 2020, 2050, and 2080) were calculated and presented in Figure 10 (the Loup watershed) and Figure 11 (the Lower Nicola River). Following Figure 10 and Figure 11, it can be concluded that despite some fluctuations in the area percentages of flood susceptibility classes, the most changes in the very low and low flood susceptibility classes were in the direction of decreasing and the majority of changes in the high and very high flood susceptibility classes were in the direction of increasing flood susceptibility in both watersheds. It is worth mentioning that the red color denoted the changes toward increasing the susceptibility (i.e., positive changes), and the blue color indicated the changes toward decreasing susceptibility (i.e., negative changes). Our findings indicated that climate change affects the flood susceptibility of watersheds, even though the changes in the flood susceptibility of watersheds are scant.

**Figure 12:**
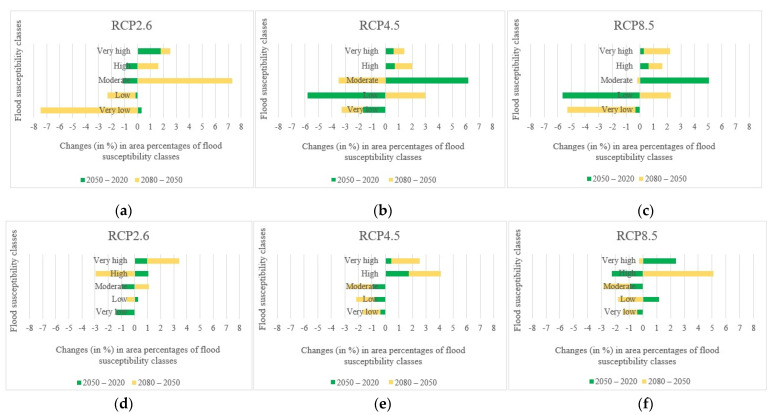
Changes (in %) in the area percentages of flood susceptibility classes over the years: (**a**) in the Loup watershed under scenario RCP2.6, (**b**) in the Loup watershed under scenario RCP4.5, (**c**) in the Loup watershed under scenario RCP8.5, (**d**) in the Lower Nicola River watershed under scenario RCP2.6, (**e**) in the Lower Nicola River watershed under scenario RCP4.5, (**f**) in the Lower Nicola River watershed under scenario RCP8.5.

The variations in area percentages of flood susceptibility classes were plotted in Figure 12 to provide a better depiction of changes throughout time. The results in Figure 12 indicated that the changes in the Loup watershed mostly happened between the years 2050 and 2080 under the climate change scenario RCP2.6 and between the years 2020 and 2050 under the climate change scenarios RCP4.5 and RCP8.5. In the Lower Nicola River watershed, on the other hand, the most changes occurred between 2050 and 2080 in all climate change scenarios.

**Figure 13:**
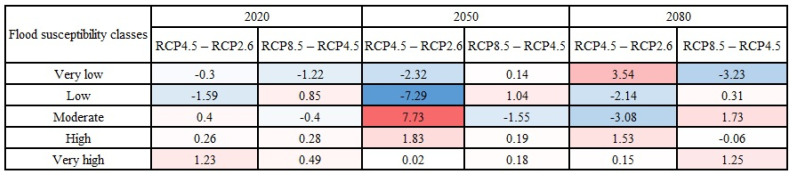
Changes (in %) in the area percentages of flood susceptibility classes based on the changes in the climate change scenarios in the Loup watershed.

**Figure 14:**
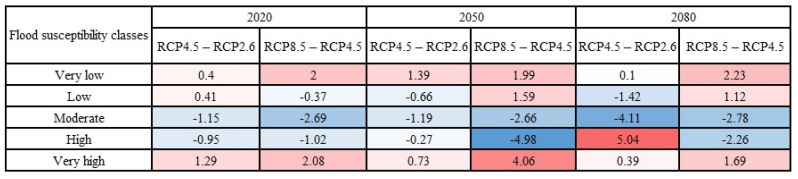
Changes (in %) in the area percentages of flood susceptibility classes based on the changes in the climate change scenarios in the Lower Nicola River watershed.

In addition to investigating changes over time, the changes in the area percentages of flood susceptibility classes were assessed depending on three climate change scenarios: optimistic (RCP2.6), business as usual (RCP4.5), and pessimistic (RCP8.5). The changes in the area percentages of flood susceptibility classes concerning the changes in the climate change scenarios in the Loup watershed and the Lower Nicola River watershed were calculated and illustrated in Figure 13 and Figure 14, respectively. As with the changes over time, despite some fluctuations in area percentages of flood susceptibility classes, most of the changes in the high and very high flood susceptibility classes in both watersheds were toward rising as the scenarios changed from RCP2.6 to RCP4.5 and from RCP4.5 to RCP8.5. Following the findings in Figure 13 and Figure 14, even though the changes in the area percentages of each flood susceptibility class seem trivial, the area percentages of flood susceptibility classes in both watersheds were affected under various climate change scenarios.

**Figure 15:**
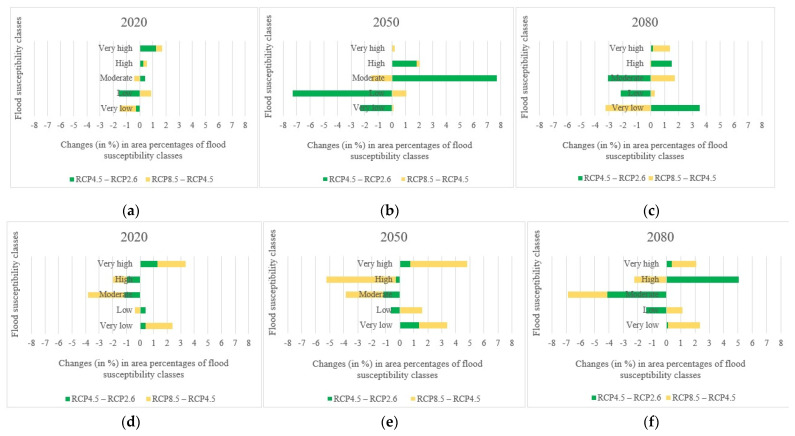
Changes (in %) in the area percentages of flood susceptibility classes regarding the changes in the climate change scenarios: (**a**) in the Loup watershed in the year 2020, (**b**) in the Loup watershed in the year 2050, (**c**) in the Loup watershed in the year 2080, (**d**) in the Lower Nicola River watershed in the year 2020, (**e**) in the Lower Nicola River watershed in the year 2050, (**f**) in the Lower Nicola River watershed in the year 2080.

To have a better representation of variations regarding the changes in the climate change scenarios, the changes in area percentages of flood susceptibility classes were plotted in Figure 15. The changes in the Loup watershed occurred mainly between the scenarios RCP2.6 and RCP4.5 in the years 2050 and 2080 and almost the same in the year 2020. The most changes in the Lower Nicola River watershed, on the other hand, occurred between RCP2.6 and RCP4.5 in 2080 and between RCP4.5 and RCP8.5 in the years 2020 and 2050.

## 4. Discussion

Floods are one of the most hazardous natural disasters that usually result in considerable loss of life and significant property damage [94]. Changing climates induced by global warming have affected the circulation patterns of the atmospheric and ocean currents and, consequently the spatial and temporal patterns of precipitation. Accordingly, changes in flood susceptibility are linked to climate changes. As the ability of the atmosphere to hold moisture increases due to global warming, more frequent and heavier precipitation events may occur, raising the peril of floods [95]. To the study by Houghton et al., (2001) [96], precipitation has increased by 0.5 to 1% every decade in much of the Northern Hemisphere’s mid-to high latitudes over the last 100 years. As a result, evaluating flood-prone zones under future precipitation circumstances is crucial for gaining a thorough knowledge of future flood susceptibility patterns.

Having the capacity to predict the spatial patterns of flooding in watersheds and assess their flood susceptibility could improve the managers’ abilities to reduce flood losses. Accordingly, the primary objective of this study was to develop various ML models to identify and predict current and future flood susceptible areas while considering the spatial and temporal impacts of climate change on floods. From a spatial perspective, there are three crucial elements in efficiently mapping flood susceptibility: (1) selection of appropriate flood explanatory factors, (2) spatial resolution of the flood explanatory factors, and (3) the accuracy and efficiency of data layer integration models [97]. Even though there is no conventional technique for selecting the factors that would best predict future floods, we chose various sets of factors regarding the literature review [5,54,55,56]. A variety of meteorological, hydrological, and geospatial flood explanatory factors were collected and prepared using RS and GIS techniques. Given the availability of data resources, various flood explanatory factors with a spatial resolution of 30 m were collected and prepared for both watersheds. Moreover, as the third substantial element in efficiently mapping flood susceptibility, four various models of MLP-NN, RF, NB, and GBM, as promising ML models, were employed to provide the flood susceptibility map.

Following the literature review, numerous ML models were formulated and developed to map flood susceptibility [27,57,60,98,99,100,101,102,103]. In this study, four potential ML models of MLP-NN, NB, RF, and GBM, were used to assess the flood susceptibility in two different watersheds in Canada. Moreover, three accuracy criteria were used to evaluate and compare the accuracy of the models. Regarding the results of three accuracy metrics, the RF model outperformed the rest of the employed models, which was consistent with the findings of other researchers who have described the RF model as a more accurate model [5,28].

After evaluating the efficiency and accuracy of the employed ML models, the model with the best accuracy was chosen and run using the precipitation data in the years 2020, 2050, and 2080 under three climate change scenarios: optimistic (RCP2.6), business as usual (RCP4.5), and pessimistic (RCP8.5). Accordingly, for each year as well as under each scenario, a flood susceptibility map was provided for both watersheds. The results of this study indicated that the flood susceptibility of both the Loup and Lower Nicola River watersheds worsens over time. Our findings were consistent with the study by Janizadeh et al., (2021) [34], demonstrating that flood susceptibility worsens over time.

Although the effects of climate change are still debatable, the impacts of climatic variability require more investigation. While precipitation is recognized as the most significant climatic factor for flooding in some places [104] and the runoff factor in flood events [105], regarding many earlier studies, the most influential factors for flood events include elevation [26,52], slope [52], distance from rivers [26,52,106,107], drainage density, and land cover/land use [52,97]. Comparing the area percentages of flood susceptibility classes in both watersheds given their corresponding precipitation maps demonstrated that even though the Loup watershed receives significantly more precipitation than the Lower Nicola River watershed, the area percentages of moderate, high, and very high flood susceptibility classes in the Loup watershed were much trivial compared to the area percentages of the same classes in the Lower Nicola River watershed. Consequently, our findings indicated that the precipitation factor single-handedly is inadequate in identifying flood-prone regions and that topographic, hydrologic, environmental, and geologic factors must be considered and investigated in addition.

Datasets in ML models are divided into training and test datasets, where the training datasets are used for training and the performance of the models are evaluated based on the test dataset. The trained model may have poor generalization in space and time if the distributions between the training and test sets change (i.e., distribution shifts) or if there are inherent sample dependencies. The in-built assumption of independent and identical distribution (I.I.D) is automatically made when data is divided into training-test sets. Accordingly, the assumption of I.I.D is central to almost all ML algorithms. However, spatial autocorrelation and spatial heterogeneity (i.e., two intrinsic properties of spatial data) violate I.I.D assumption [108]. Due to the increased resemblance between neighboring data samples, spatial autocorrelation violates the independence principle. On the other hand, because the data generating processes frequently change with respect to space, spatial heterogeneity violates the identical distribution assumption [109]. To overcome spatial autocorrelation and spatial heterogeneity issues, the process of choosing flood and non-flood points was done iteratively and the set of points which the *p*-value of Moran’s I for all factors was obtained extremely close to zero and less than the threshold (i.e., 0.05) was chosen to create the flood inventory map.

Although ML models have produced encouraging results in flood forecasting, essential uncertainties exist in their prediction results. Regardless of the ML model employed, numerous error sources influence the prediction results. The errors come from at least three significant sources. First, not only ML models but also all computational models are amplifications and approximations of a complex physical system due to mathematical and modeling restrictions. Second, ML models learn to extract patterns from the input data; therefore, training models with insufficient quality or scarce data will result in uncertainty in model predictions. Third, there will be more uncertainty since we cannot predict how the properties of real system could alter in the future. When extrapolating from the past to the future as is customary, uncertainty arises from both the imprecise depiction of the past and the degree to which the future will resemble the past [110].

### Strengths and Limitations

The strengths of this study include: (i) considering 16 various meteorological, topographic, hydrologic, environmental, and geologic flood conditioning factors in the flood susceptibility assessment, (ii) using four various ML models, (iii) considering two alternative watersheds with diverse meteorological, topographic, hydrologic, environmental, and geologic conditions and comparing the flood susceptibility of them with each other, (iv) evaluating and comparing the performance of ML methods using three various accuracy criteria, and (v) evaluating the flood susceptibility in three years and under three climate change scenarios: optimistic (RCP2.6), business as usual (RCP4.5), and pessimistic (RCP8.5).

Notwithstanding these strengths, this study had few limitations in terms of future precipitation data and the preparation process of them. Future precipitation data have uncertainty and include some level of errors. On the other hand, the interpolation process also imports some level of errors in the data. Another limitation of this study was considering only precipitation as the dynamic flood explanatory factor following the primary goal of the study, while other factors such as land cover also vary over time. The creation of the flood inventory map for a single flood occurrence was another research constraint. A single flood event was considered in both study areas due to a paucity of data on previous flood occurrences in the study areas.

## 5. Conclusions

Flooding is one of the most devastating natural disasters that can result in death, injury, property destruction, loss of livelihoods and services, social and economic upheaval, and environmental havoc. Climate change has the potential to exacerbate the runoff rates and patterns and the hydrological cycle, resulting in more intense precipitation and increases in flood intensity, frequency, and severity.

In this study, four various ML models, including MLP-NN, NB, RF, and GBM, were used to provide the current and future flood susceptibility maps in two different watersheds in Canada, one in Quebec province and the other in British Columbia province. Moreover, three RCP2.6, RCP4.5, and RCP8.5 climate change scenarios were examined to address the implications of climate change.

Regarding the accuracy metrics, the RF model had the highest accuracy and was chosen as the best ML model to provide the flood susceptibility maps. Regarding the provided flood susceptibility maps, flooding is more likely in the Lower Nicola River watershed than in the Loup watershed. The most flood-prone locations in the Loup watershed were in the southern and southeast, while the most flood-prone areas in the Lower Nicola River watershed were in the center, northeast, and northwest. The results of this study indicated that the flood susceptibility of both the Loup and Lower Nicola River watersheds worsens over time.

The contribution of this study lies in the identification of flood-prone areas over time and under various climate change scenarios in two different watersheds. The spatial forecasts provided by this research aim to assist disaster management agencies in making critical decisions and contributions to mitigate the damages caused by floods, and to better inform local communities and researchers on the important role and influence of climate change on flood susceptibility.

## Figures and Tables

**Table 1:** Flood explanatory data along with their sources.

Primary Input Data	Original Format Sources	Source	Derived Map
Shuttle Radar Topography Mission (SRTM); DEM	Raster	United States Geological Survey (USGS); https://earthexplorer.usgs.gov/ (accessed on 1 March 2022)	Elevation
			Slope
			Aspect
			Plan curvature
			Profile curvature
			SPI
			TWI
			Roughness
Land cover map	Vector (i.e., polygons)	Government of Canada, Natural resources Canada; https://open.canada.ca/data/en/dataset/4e615eae-b90c-420b-adee-2ca35896caf6 (accessed on 1 March 2022)	Land cover map
Climatological stations	Vector (i.e., points)	Government of Canada, Environment and natural resources; https://climate.weather.gc.ca/historical_data/search_historic_data_e.html (accessed on 1 March 2022) and Climate Data Canada; https://climatedata.ca/ (accessed on 1 March 2022)	Precipitation map
Meteorological data	Numerical data		
Streams, Rivers, and water bodies	Vector (i.e., polylines)	Government of Canada, Statistics Canada; https://open.canada.ca/data/en/dataset/448ec403-6635-456b-8ced-d3ac24143add (accessed on 1 March 2022)	Distance from rivers
			Drainage density
Geological map	Vector (i.e., polygons)	Government of Canada, Natural resources Canada, Geological Survey of Canada; https://geoscan.nrcan.gc.ca/starweb/geoscan/servlet.starweb?path=geoscan/downloade.web&search1=R=295462 (accessed on 1 March 2022)	Lithological map
Soil map	Vector (i.e., polygons)	Government of Canada, Agriculture and Agri-Food Canada; https://open.canada.ca/data/en/dataset/5ad5e20c-f2bb-497d-a2a2-440eec6e10cd (accessed on 1 March 2022)	Soil map
Landsat 8 Operational Land Imager (OLI) and Thermal Infrared Sensor (TIRS)	Raster	United States Geological Survey (USGS); https://earthexplorer.usgs.gov/ (accessed on 1 March 2022)	NDVI
			NDMI

**Table 2:** VIF and Tolerance values in multi-collinearity analysis for all flood explanatory factors in both watersheds.

Predictors/Factors	Collinearity Statistics in the Loup Watershed		Collinearity Statistics in the Lower Nicola River Watershed	
	Tolerance	VIF	Tolerance	VIF
SPI	0.735	1.360	0.632	1.581
TWI	0.344	2.906	0.383	2.612
Precipitation	0.228	4.388	0.680	1.470
Drainage density	0.388	2.575	0.368	2.718
Distance from rivers	0.379	2.641	0.413	2.419
Lithology	0.356	2.811	0.805	1.242
Soil	0.511	1.955	0.578	1.731
Land cover	0.358	2.790	0.824	1.213
NDVI	0.231	4.338	0.458	2.182
NDMI	0.350	2.858	0.615	1.626
Roughness	0.444	2.254	0.856	1.168
Plan curvature	0.573	1.746	0.622	1.607
Profile curvature	0.572	1.747	0.663	1.509
Aspect	0.760	1.316	0.928	1.077
Slope	0.571	1.752	0.472	2.119
DEM	0.164	6.107	0.338	2.956

**Table 3:** The ROC-AUC value achieved for the training and validation dataset using various ML models.

ML Models	The Loup Watershed		The Lower Nicola River Watershed	
	Training	Validation	Training	Validation
RF	1.0	1.0	1.0	0.9968
NB	0.9991	0.9805	0.9776	0.9571
MLP-NN	0.9644	0.9614	0.8817	0.8503
GBM	1.0	0.9978	1.0	0.9967

**Table 4:** The calculated values of three accuracy metrics for each ML model.

ML Models	The Loup Watershed			The Lower Nicola River Watershed		
	ROC-AUC	FOM	F1 Score	ROC-AUC	FOM	F1 Score
RF	0.9992	0.9767	0.9882	0.9996	0.9787	0.9892
NB	0.9548	0.8864	0.9398	0.9884	0.8036	0.8911
MLP-NN	0.9643	0.7857	0.88	0.9411	0.6458	0.7848
GBM	0.9901	0.9333	0.9655	0.9979	0.9787	0.9892

## Data Availability

All the data utilized in this research are openly accessible (at the time of writing this manuscript).
